# Insulin and the insulin receptor collaborate to promote human gastric cancer

**DOI:** 10.1007/s10120-021-01236-y

**Published:** 2021-09-23

**Authors:** Marina Saisana, S. Michael Griffin, Felicity E. B. May

**Affiliations:** 1 Newcastle University Centre for Cancer, Translational and Clinical Research Institute, Faculty of Medical Sciences, University of Newcastle-upon-Tyne, Framlington Place, Newcastle-upon-Tyne, NE2 4HH UK; 2 Department of Surgery, Newcastle-upon-Tyne Hospitals NHS Foundation Trust, Newcastle-upon-Tyne, NE1 4LP UK; 3 Department of Pathology, Faculty of Medical Sciences, University of Newcastle-upon-Tyne, Framlington Place, Newcastle-upon-Tyne, NE2 4HH UK; 4 Department of Oncology, Newcastle-upon-Tyne Hospitals NHS Foundation Trust, Newcastle-upon-Tyne, NE1 4LP UK

**Keywords:** Insulin, Insulin receptor, Gastric cancer, Obesity, Insulin-like growth factor

## Abstract

**Background:**

Gastric adenocarcinoma is common and consequent mortality high. Presentation and mortality are increased in obese individuals, many of whom have elevated circulating insulin concentrations. High plasma insulin concentrations may promote, and increase mortality from, gastric adenocarcinoma. Tumour promotion activities of insulin and its receptor are untested in gastric cancer cells.

**Methods:**

Tumour gene amplification and expression were computed from sequencing and microarray data. Associations with patient survival were assessed. Insulin-dependent signal transduction, growth, apoptosis and anoikis were analysed in metastatic cells from gastric adenocarcinoma patients and in cell lines. Receptor involvement was tested by pharmacological inhibition and genetic knockdown. RNA was analysed by RT-PCR and proteins by western transfer and immunofluorescence.

**Results:**

*INSR* expression was higher in tumour than in normal gastric tissue. High tumour expression was associated with worse patient survival. Insulin receptor was detected readily in metastatic gastric adenocarcinoma cells and cell lines. Isoforms B and A were expressed. Pharmacological inhibition prevented cell growth and division, and induced caspase-dependent cell death. Rare tumour *INS* expression indicated tumours would be responsive to pancreatic or therapeutic insulins. Insulin stimulated gastric adenocarcinoma cell PI3-kinase/Akt signal transduction, proliferation, and survival. Insulin receptor knockdown inhibited proliferation and induced programmed cell death. Type I IGF receptor knockdown did not induce cell death.

**Conclusions:**

The insulin and IGF signal transduction pathway is dominant in gastric adenocarcinoma. Gastric adenocarcinoma cell survival depends upon insulin receptor. That insulin has direct cancer-promoting effects on tumour cells has implications for clinical management of obese and diabetic cancer patients.

**Supplementary Information:**

The online version contains supplementary material available at 10.1007/s10120-021-01236-y.

## Introduction

Gastric cancer is the second leading cause of cancer-related death worldwide; 783,000 patients die annually [1]. Many patients present with incurable, late-stage disease and systemic treatment options are limited [2]. Genomic studies identified gene amplification of *ERBB2*, *EGFR*, *FGFR2*, *MET* and *IGF1R*, that encode targetable tyrosine kinase receptors [3] and activating mutations in *KRAS*, *BRAF* [4] and *PI3KCA*. [3] Gastric adenocarcinoma patients with advanced disease and amplified *ERBB2* are eligible for trastuzumab therapy [5]. Otherwise, targeted therapies have had relatively little impact. Phase II clinical trials of drugs against FGFR2 and c-Met were promising but phase III trials disappointed [6]. The majority of gastric adenocarcinoma patients, around 70%, would be ineligible for these targeted agents [7].

Epidemiological studies indicate that insulin promotes higher rates of gastric cancer presentation and mortality. Obesity is associated positively with gastric cancer incidence and mortality [8, 9]. Obesity leads to insulin resistance, compensatory hyperinsulinemia, metabolic syndrome and type II diabetes mellitus [10]. High therapeutic insulin doses are required to overcome insulin resistance in type II diabetes. Preceding hyperinsulinemia and high therapeutic insulin concentrations could explain the prevalence of gastric cancer in type II diabetics [11, 12]. There is a positive association between plasma insulin levels and gastric cancer incidence [13]. Treatment of type II diabetes with metformin, which lowers insulin secretion [14], reduces gastric cancer incidence [15, 16] Type I diabetics have also increased gastric cancer incidence [17, 18], that reaches 3.3-fold after 15 years of insulin therapy [17].

The insulin signal transduction pathway is activated by three ligands, insulin, insulin-like growth factor 1 (IGF-1) and insulin-like growth factor 2 (IGF-2) via interaction with two closely-related tyrosine kinase receptors. [10, 19] The insulin and type I IGF receptors signal through common downstream intracellular pathways. The insulin receptor has two isoforms [20]. Isoform B is present across species whereas isoform A, which is expressed in foetal cells, is found uniquely in mammals [21].

Despite epidemiological evidence that high circulating insulin concentrations, secreted by the pancreas in overweight individuals or injected to treat diabetes, promote gastric cancer and increase ensuant mortality, the possibility that insulin has direct tumour-promoting effects upon the malignant cells themselves has not been tested. Objectives were to investigate if insulin promotes directly gastric adenocarcinoma cell proliferation or survival, and to explore the relative importance of the insulin receptor.

## Materials and methods

### Isolation of metastatic cells

Ethical permission was obtained from the Joint Newcastle Health Hospitals and University of Newcastle-upon-Tyne Ethical Committee to obtain metastatic patient samples for ex vivo analyses. All patients gave informed consent. Ascitic fluid rich in disseminated tumour cells, was drained from the peritoneal cavities of patients with advanced gastric adenocarcinoma (Table 1). The cell suspension was diluted 1:1 with Dulbecco’s modified Eagle’s medium (DMEM), 20% untreated foetal calf serum (FCS) and 1% penicillin/streptomycin (Sigma) and maintained at 37 °C.

**Table 1:** Clinicopathological features of patients from whom metastatic cells were isolated

Patient ID	Sex	Age (years)	Diagnosis	Stage at diagnosis	Systemic treatment	HER2 status primary	HER2 status disseminated cells
GC1	Male	~ 65	Gastric adenocarcinoma	T4N2M1	Symptom control with palliative drain	N.D	Negative
JW1	Male	75	Gastric adenocarcinoma, poorly differentiated	T4N1M1	Palliative radiotherapy, 30 Gy in 10 fractions	N.D	Negative
HC1	Female	68	Gastric adenocarcinoma, diffuse type, linus plastica	T3N1M0	Three cycles of peri-operative ECX	N.D	Negative
NC1	Female	52	Gastric adenocarcinoma, of the cardia	T4N2M1	Symptom control with palliative drain	Negative	Negative

### Cell culture

Gastric adenocarcinoma cell lines were purchased from ATCC. NCI-N87, MKN74, NUGC3, AGS were cultured as adherent monolayers, SNU-1, SNU-5 and SNU-16 in suspension and KATO III as a mix of adherent and suspension cells in DMEM with 10% untreated FCS or 20% untreated FCS (SNU-5). NCI-N87 have amplified *ERBB2*, KATO III and SNU-16 have amplified *FGFR2* and SNU-5 have amplified *MET*. SNU-1, MKN74, NUGC3 and AGS are triple-negative for amplification of these three targetable tyrosine kinase receptor oncogenes [7]. SNU-1 have activated *KRAS-G12D*, MKN74 have impaired *BRAF-G466V* and AGS have activated *KRAS-G12D* and *PIK3CA-E453K/E545A * [7]. Cells were tested to confirm absence of mycoplasma and 100% authenticity confirmed by STR analysis.

### Stimulation of phosphorylation

Cells were placed in 22 mm-diameter wells at 100,000–150,000 cells/well, incubated for 24–48 h, washed with phosphate buffered serum pH 7.2 (PBS) and withdrawn from stimulation by growth and survival factors that are present in serum by culture in withdrawal medium for one (SNU-1) or two days. Withdrawal medium comprised phenol red-free DMEM and 10% calf serum that had been incubated with dextran-coated charcoal at 55 °C to remove steroids and growth and survival factors [22, 23]. Cells were washed and incubated with serum-free, phenol red-free DMEM and 0.1% bovine serum albumin (BSA; Sigma-Aldrich) at 37 °C for 2 h followed by the same serum-free medium plus insulin, IGF-1 or IGF-2 for 15 min—1 h. Cells were incubated with BMS-754807, for 30 min before addition of ligand.

### Western transfer

Cells were lysed in radioimmunoprecipitate (RIPA) buffer and analysed by western transfer as described [24] with antibodies against: insulin receptor (#3025), type I IGF receptor (#3027), β-tubulin (#5346), phosphorylated Tyr 1150/1151 insulin and Tyr 1135/1136 type I IGF receptors (#3024), Akt (#9272), phosphorylated Ser 473 Akt (#4060), ERK1 and ERK2 (#9102), phosphorylated Thr 202/204 ERK1 and Thr 185/187 ERK2 (#4370), cleaved Asp 214 poly-(ADP-ribose) polymerase-1 (PARP-1) (#9541) (Cell Signaling Technologies), or GAPDH-HRP (sc-25778) (Santa Cruz Biotechnology). Protein bands were quantified by densitometry and adjusted for GAPDH, β-tubulin or the corresponding total protein [24].

### Apoptosis

Between 100,000 and 150,000 cells were added to 22 mm-diameter wells and incubated for 24 h. Cells were withdrawn from growth and survival factors in serum by culture in withdrawal medium for one (SNU-1) or two days [22, 24]. Cells were incubated with 0–200 ngml^−1^ of insulin, IGF-1 or IGF-2 in withdrawal medium for 15 min before treatment with 0.5–1 μM staurosporine (Sigma-Aldrich, Dorset, United Kingdom) for 4–5 h. Alternatively, cells were cultured in DMEM with untreated FCS and different concentrations of BMS-754807.

For anoikis, culture vessels were coated with poly(hydroxyethyl methacrylic) (poly-HEMA; SIGMA) which inhibits matrix deposition and hence cell attachment [7, 24]. Cells were added to 35 mm-diameter poly-HEMA-coated wells at 400,000 cells/well and cultured in serum-free medium in the absence or presence of ligand or BMS-754807 for 24 h [24].

### siRNA knockdown

Double-stranded, short-interfering RNA sequences against insulin receptor and type I IGF receptor, and scrambled sequences were purchased from Sigma-Aldrich. Oligonucleotides and lipofectamine (Invitrogen, Paisley, United Kingdom) were pre-incubated for 30 min at room temperature and added to cells in suspension in the presence of untreated FCS. Cells were plated at 50,000 cells per 1.9 cm^2^ and incubated for ≥ 24 h in untreated FCS-containing medium.

### Immunofluorescence

Cells were fixed in methanol, 70% ethanol or 4% paraformaldehyde and incubated with Alexa Fluor-conjugated antibodies against cleaved Asp 175 caspase-3 (#9603), cleaved Asp 214 PARP-1 (#6894), or unconjugated-antibodies against BrdU (#5292) or phosphorylated Ser 10 histone H3 [25] (#9701) (Cell Signaling Technologies), followed by Alexa Fluor-conjugated secondary antibodies (#A-11034, #A-11001) (Invitrogen). For BrdU incorporation, cells were treated with 0.03 mg/ml BrdU for 2 h before and 1.5 M HCl for 30 min after fixation. Cells were mounted in Vectashield with DAPI (Vector Laboratories).

### Proliferation

Cells were added to 16 mm-diameter wells at 5000–10,000 cells/well, cultured in withdrawal medium for 24 h and then in withdrawal medium minus and plus 50 ng/ml insulin, IGF-1 or IGF-2 for 0, 3, 6, or 9 days. Alternatively, cells were cultured in untreated FCS-containing medium and different BMS-754807 concentrations. Cells were lysed in 0.15 M NaCl, 15 mM sodium citrate, pH 7.0, and 0.02% SDS at 37 °C for 30 min. DNA was sheared and measured with PicoGreen dsDNA Quantitation Reagent (Invitrogen) [7, 23].

### Reverse transcription polymerase chain reaction (RT-PCR)

RNA was extracted with Direct-zol™ RNA MiniPrep Kit (Zymo Research, California). cDNA was synthesised from 1 µg RNA with Moloney Murine Leukaemia Virus Reverse Transcriptase (USB) in a 20 μl reaction and 0.1 μl cDNA amplified with Red Hot DNA polymerase (Thermo-scientific) in 10 μl [26, 27]. Primers designed with Primer-BLAST were synthesised by SIGMA-Aldrich. Amplicons were analysed on 3% agarose gels, stained with GelRed (Cambridge Bioscience, Cambridge, UK).

### Patient cohort analyses and statistics

For survival analyses, tumour gene expression was measured by Affymtrix array hybridisation in 882 patients [28] and by RNA-Seq in 392 patients [29]. Copy number variation (CNV) and gene expression data deduced by genomic and RNA sequencing in 478 gastric tumours were obtained from the Cancer Genome Atlas (TCGA) and analysed. Gene expression was analysed by RNA-Seq in 34 normal gastric tissue samples and in 33 tumour types [29], and by microarray analysis in 1065 gastric adenocarcinomas [28].

Associations between copy number and expression were analysed by Kruskal–Wallis one-way ANOVA with Dunn’s post hoc comparison between groups and Boniferroni corrections. Associations between gene expression and overall survival were analysed by the log rank test [28]. Differences between groups were tested by student’s t-test, or one-way or two-way analysis of variance (ANOVA). Differences between experimental conditions were tested by Dunnett’s or Tukey’s multiple comparisons tests.

Representative images of experiments are shown. Triplicate or more samples were analysed. Normalized data are expressed as a percentage of the maximum effect. Results are shown as mean ± S.E.M. Results of statistical analyses are detailed in the legends to the figures. Differences were considered statistically significantly if *p* < 0.05.

## Results

### Expression of *INSR *and *IGF1R* in gastric adenocarcinoma

**Fig. 1:**
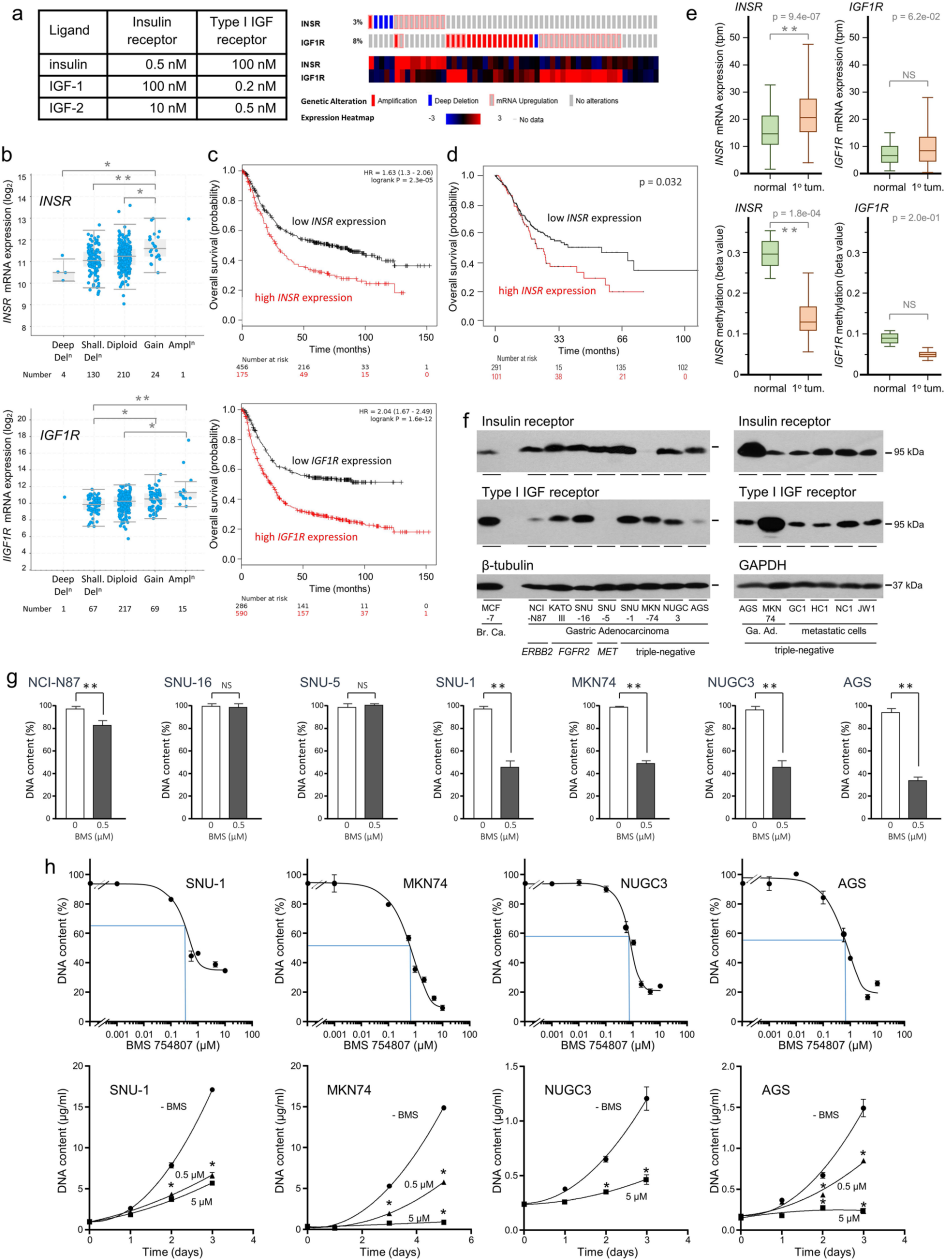
Expression of genes that encode, and inhibition of, the insulin and type I IGF receptors in gastric adenocarcinoma. **a** The affinities of insulin, IGF-1 and IGF-2 for the insulin and type I IGF receptors are shown (adapted from [10]). Original references are included in the electronic supplementary material (Online Resource 1). Copy number and relative high expression of  > 2 SD from the mean for diploid tumours of the genes that encode the insulin receptor (*INSR*) and type I IGF receptor (*IGF1R*) were analysed for 478 gastric adenocarcinomas [49, 50]. **b** Log_2_ transformed mRNA relative abundance deduced from absolute transcript abundance by RNA-Seq expectation maximization (RSEM) [51] is shown against the putative copy-number alterations deduced from genomic sequencing data analysed with GISTIC 2.0. Horizontal bars represent the median values, boxes the range of the second and third quartiles of the data and whiskers the range of all data. Expression of *INSR* and *IGF1R*, is associated with CNV (Kruskal Wallis; *p* < 0.0001); statistically significant differences between groups are indicated (Dunn-Boniferroni post hoc; *p < 0.05 (*INSR*); *p < 0.005 (*IGF1R*); **p < 0.0001 (either)). **c** Kaplan and Meier curves illustrate correlations analysed by the log rank test between *INSR* and *IGF1R* expression, and overall survival for 876 gastric cancer patients [28]. **d** Kaplan and Meier curves illustrate correlation analysed by the log rank test between *INSR* expression, and overall survival for 392 gastric cancer patients [29]. **e** *INSR* and *IGF1R* expression calculated as transcripts per million (tpm) in normal (*n* = 34) and primary gastric tumour (1° tum.; *n* = 415) tissue (Student’s *t*-test; *p* < 0.0001 and *p* = 0.06, respectively) and methylation status upstream of *INSR* and *IGF1R* transcription start sites (Student’s *t*-test; *p* < 0.0002 and *p* = 0.195, respectively). **f** MCF-7 breast cancer cells (Br. Ca.), NCI-N87, KATO III, SNU-16, SNU-5, SNU-1, MKN74, NUGC3 and AGS gastric adenocarcinoma cells (Ga. Ad.) and metastatic cells isolated from gastric adenocarcinoma patients, GC1, HC1, NC1 and JW1, were lysed and insulin receptor, type I IGF receptor, β-tubulin or GAPDH were analysed by western transfer. *ERBB2*, *FGFR2* and *MET* are amplified and overexpressed in NCI-N87, KATO III and SNU-16, and SNU-5, respectively. SNU-1, MKN74, NUGC3 and AGS and the metastatic patient cells are triple-negative for amplification or overexpression of these oncogenes [7]. **g** NCI-N87, SNU-16, SNU-5, SNU-1, MKN-74, NUGC3 and AGS were incubated in full, untreated FCS-containing medium without and with 0.5 µM BMS-754807 for three days, lysed and their DNA content measured. Asterisks indicate if there was significantly less DNA in the presence of BMS-754807 than in its absence (Student’s *t*-test; *p* < 0.01). **h** SNU-1, MKN-74, NUGC3 and AGS were incubated in untreated FCS-containing medium and different concentrations of BMS-754807 for three days or as indicated and their DNA content measured. The relative IC_50_ values are indicated by blue lines. Asterisks indicate times at which there was significantly less DNA in the presence of BMS-754807 than in its absence (one-way ANOVA; *p* < 0.0001)

Insulin, IGF-1 and IGF-2 interact with and activate the insulin and type I IGF receptors. But insulin has much higher affinity for the insulin receptor (Fig. 1a), IGF-1 has higher affinity for the type I IGF receptor, whereas IGF-2 has more similar affinities for both receptors [10]. The *INSR* and *IGF1R* genes, which encode the insulin and type I IGF receptors, were expressed in all gastric adenocarcinomas (Fig. 1b); median relative mRNA abundances were around 10 to 12 (log_2_). Amplification, deletion or relative overexpression of *INSR* and *IGF1R* were detected in 3% and 8% of gastric adenocarcinomas, respectively (Fig. 1a). Although amplified rarely, *INSR* expression, and that of *IGF1R*, was associated strongly with CNV (Kruskal Wallis; *p* < 0.0001). (Fig. 1b).

In terms of patient outcome, higher tumour *INSR* expression, and *IGF1R* expression, was associated significantly with shorter overall survival (Fig. 1c). The significant association between high *INSR* expression and poor survival was corroborated in a separate cohort of 392 patients (Fig. 1d) [29]. Expression of *INSR,* but not *IGF1R,* was significantly higher in tumour than in normal gastric tissue (Fig. 1e). There was some indication that reduced promoter methylation might contribute to higher tumour *INSR* expression.

Receptor levels were analysed in gastric adenocarcinoma cell lines and metastatic cells isolated from the peritoneal cavity of advanced gastric cancer patients (Table 1). *ERBB2* is amplified and overexpressed in NCI-N87, *FGFR2* in KATO III and SNU-16, and *MET* in SNU-5. SNU-1, MKN74, NUGC3, AGS and the metastatic patient cells are triple-negative for amplification or overexpression of these three oncogenes [7]. Consistent with relatively high tumour *INSR* expression, insulin receptor was detected readily in seven of eight cell lines, at lower levels in MKN74 and in metastatic cells (Fig. 1f). Type I IGF receptor was detected as reported previously [7]. Notably, insulin receptor levels are visualised easily in three of the four gastric cancer cell lines and in the metastatic cells that represent the majority of gastric cancers that are triple-negative for *ERBB2*, *FGFR2* or *MET* overexpression [7]. Similar levels of insulin receptor were detected in NCI-N87, Kato III and SNU-16, and SNU-5 that have amplification and overexpression of *ERBB2*, *FGFR2*, and *MET*, respectively [7].

### Pharmacological inhibition of insulin and type I IGF receptors

The importance of the insulin and IGF signal transduction pathway was tested with BMS-754807 a specific dual inhibitor of the insulin and type I IGF receptors in gastric adenocarcinoma cells exposed to growth factors present in untreated FCS. There was a reduction in DNA content in NCI-N87, no change in SNU-16 or SNU-5 and a marked reduction in SNU-1, MKN74, NUGC3 and AGS (Fig. 1g). Receptor inhibition was more effective in gastric adenocarcinoma cells that are triple-negative for amplification or overexpression of *ERBB2*, *FGFR2* or *MET*. Subsequent experiments focused on triple-negative cancer.

Incubation of SNU-1, MKN74, NUGC3 and AGS with different concentrations of BMS-754807 in the presence of untreated FCS demonstrated concentration-dependent reduction in DNA content (Fig. 1h). Relative IC_50_ values were between 0.35 and 0.7 μM BMS-754807. Over time, cell growth was reduced by 0.5 μM BMS-754807 in SNU-1, MKN74 and AGS and by 5 μM BMS-754807 in all cells (two-way ANOVA; *p* < 0.0002).

**Fig. 2:**
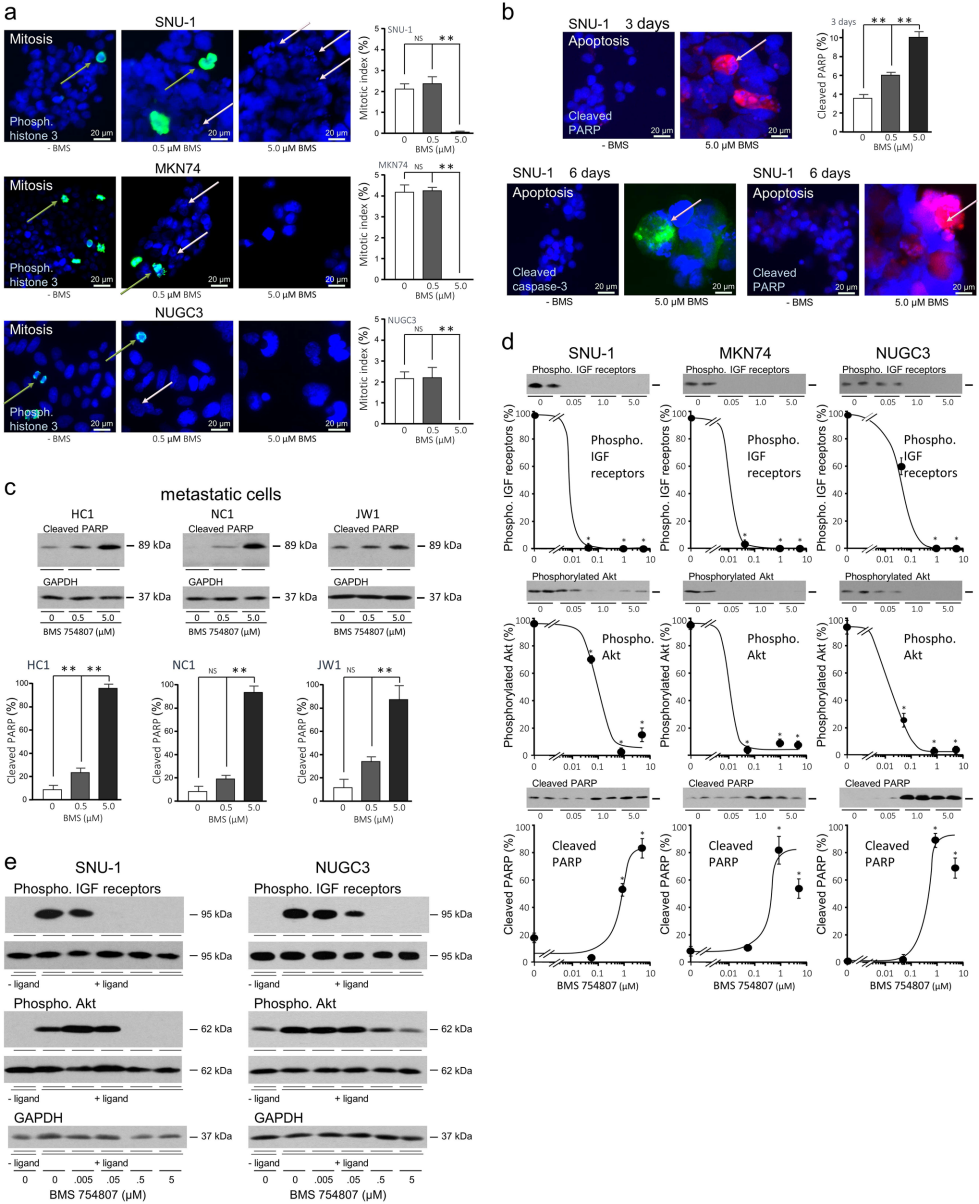
Pharmacological inhibition of the insulin and type I receptors induces cell death **a** SNU-1 were cultured in suspension, whereas MKN74 and NUGC3 were plated onto coverslips, in untreated FCS-containing medium and the indicated concentration of BMS-754807 for three days. Cells were assayed for histone H3 Ser 10 phosphorylation (khaki arrows) by immunofluorescence and counterstained with DAPI. Asterisks indicate that the proportion of cells in the mitotic-phase of the cell cycle is significantly lower after incubation with BMS-754807 (one-way ANOVA; SNU-1, *p* = 0.0009; MKN74, *p* = 0.0046; NUGC3, *p* = 0.0023). NS indicates data that are not significantly different. Pale pink arrows indicate apoptotic cells. **b** SNU-1 were incubated in untreated FCS-containing with BMS-754807 (BMS) for three or six days. Cleaved caspase-3 or cleaved PARP were analysed by immunofluorescence (pink arrows). Asterisks indicate that the proportion of cells with detectable cleaved PARP is statistically significantly different after incubation with BMS-754807 (one-way ANOVA; *p* < 0.01). **c** Metastatic adenocarcinoma cells were isolated from patients and incubated in 20% untreated FCS-containing medium and different concentrations of BMS-754807 for three days after which cleaved PARP and GAPDH were analysed by western transfer. The protein bands were quantified by densitometry and corrected for GAPDH. Asterisks indicate concentrations of BMS-754807 at which there was statistically significantly more cleaved PARP in its presence than in its absence (one-way ANOVA; *p* < 0.001). **d** Cells were incubated in full, untreated FCS-containing medium with BMS-754807 for three days and phosphorylated (Phospho.) insulin Tyr 1150/1151 and type I IGF Tyr 1135/1136 receptors (IGF receptors), Akt Ser 473 and cleaved PARP analysed as above, quantified  and corrected for corresponding total protein or GAPDH, respectively. Representative images are shown as inserts. Asterisks indicate concentrations of BMS-754807 at which there was statistically significantly less phosphorylated protein, or more cleaved PARP than in its absence (one-way ANOVA; *p* < 0.001). **e** SNU-1 and NUGC3 were incubated in serum-free medium for two hours and then in serum-free medium and the indicated concentrations of BMS-754807 in the absence or presence of ligand (lig.) for 15 min. Phosphorylated (Phospho.) insulin Tyr 1150/1151 and type I IGF Tyr 1135/1136 receptors (IGF receptors), Akt Ser 473 or GAPDH were measured. Total corresponding protein is shown underneath the phosphorylated protein images

To assess effects of BMS-754807 on cell division, phosphorylation of histone H3 Ser10 which occurs during chromosome condensation in early mitosis [25] was analysed by immunofluorescence. Mitotic SNU-1, MKN74 and NUGC3 were detected after culture in untreated-FCS-containing medium (Fig. 2a), but not after addition of 5 µM BMS-754807. Pharmacological inhibition of the insulin and type I IGF receptors prevented serum-stimulated gastric adenocarcinoma cell division.

Nuclear fragmentation indicative of apoptosis was visible in some SNU-1, MKN74 and NUGC3 after treatment with 0.5 µM BMS-754807 and in some SNU-1 after treatment with 5 µM BMS-754807 (Fig. 2a). Induction of programmed cell death was confirmed by detection of cleaved PARP, a product of the executioner caspase-3 after three days, and of activated, cleaved caspase-3 and cleaved PARP after 6 days’ treatment with BMS-754807 in the presence of FCS (Fig. 2b).

We investigated if pharmacological inhibition induced programmed cell death in metastatic gastric adenocarcinoma cells isolated from patients (Table 1). More cleaved PARP was detected after three days treatment with BMS-754807 in the full complement of growth and survival factors present in untreated FCS (Fig. 2c) (one-way ANOVA; *p* < 0.001), consistent with the insulin and IGF signal transduction pathway providing a dominant cell survival signal in these metastatic cells.

Concentration-dependent prevention by BMS-754807 of untreated FCS-stimulated IGF receptor phosphorylation and reduced activation of the PI3-kinase/Akt pathway was demonstrated in SNU-1, MKN74 and NUGC3 gastric adenocarcinoma cells (Fig. 2d). PARP cleavage indicative of programmed cell death was induced by 0.5 and 5.0 µM BMS-754807 (Fig. 2d). Effective inhibition of ligand-stimulated phosphorylation of IGF receptors and Akt by BMS-754807 in serum-free medium was confirmed in SNU-1 and NUGC3 (Fig. 2e).

### Expression of *INS*, *IGF1 *and *IGF2*

**Fig. 3:**
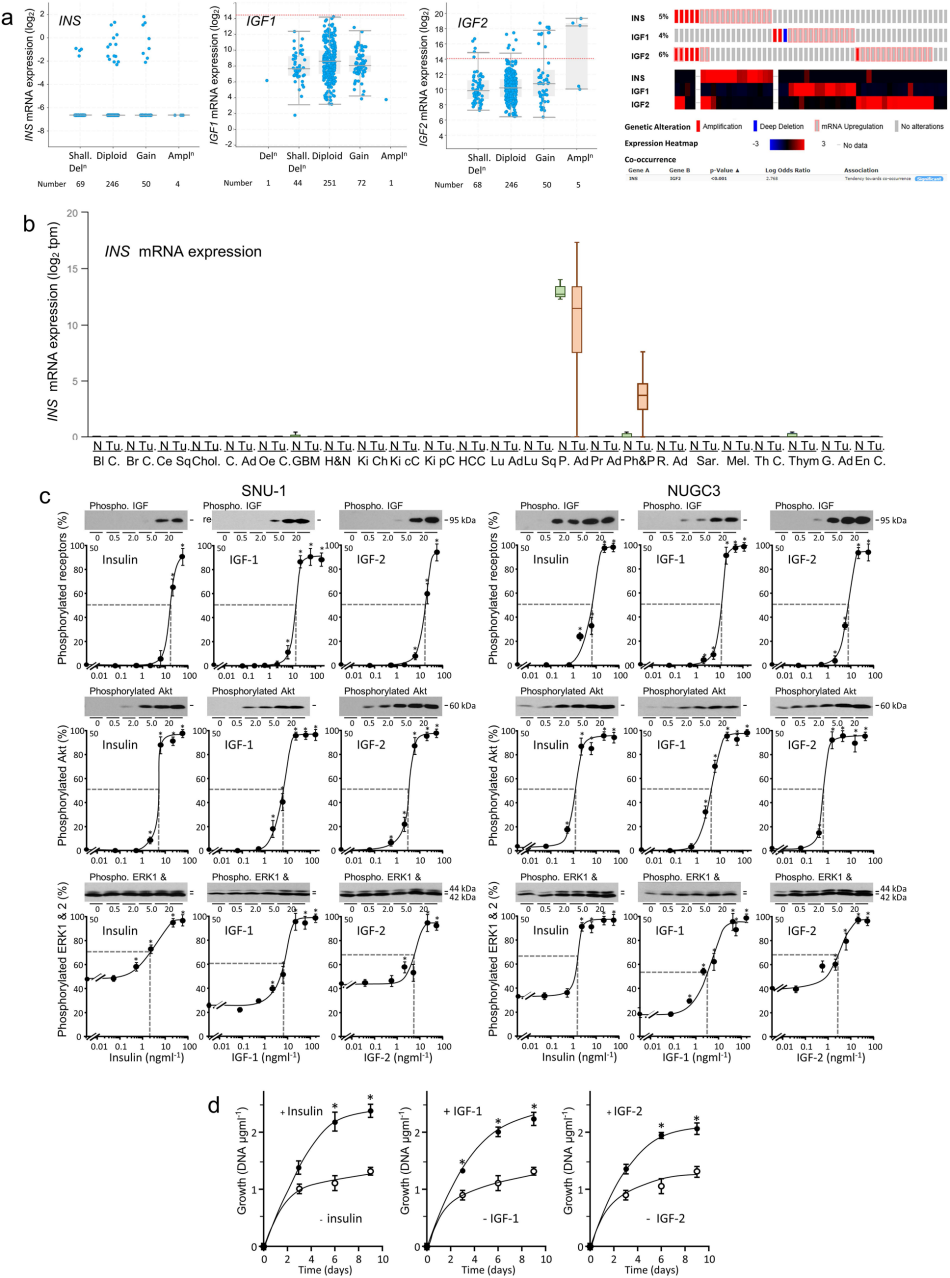
Expression of the genes that encode, and activity of, the three IGF ligands in human gastric adenocarcinomas. **a** Expression of the genes that encode insulin (*INS*), IGF-1 (*IGF1*) and IGF-2 (*IGF2*) in gastric adenocarcinomas presented as log_2_ transformed relative mRNA abundance [51] is shown against copy-number alterations as described in the legend to Fig. 1. The horizontal, brick red dotted lines indicate relative mRNA abundances of 20,000 (14.29 log_2_). Expression of *INS* and *IGF1* is not, but of *IGF2* is weakly, associated with CNV (Kruskal Wallis; *p* < 0.05). Copy number and relative high expression of *INS*, *IGF1* and *IGF2* were analysed in gastric adenocarcinomas as described in the legend to Fig. 1. **b** Expression of *INS* in normal (N) and tumour (Tu.) tissue of bladder carcinoma (Bl C.), breast carcinoma (Br C.), cervical squamous cell carcinoma (Ce Sq), cholangiocarcinoma (Chol.), colon adenocarcinoma (C. Ad), oesophageal carcinoma (Oe. C.), glioblastoma (GBM), head and neck squamous cell carcinoma (H&N), kidney chromophobe (Ki Ch), kidney clear cell carcinoma (Ki cC), kidney papillary cell carcinoma (Ki pC), hepatocellular carcinoma (HCC), lung adenocarcinoma (Lu C.), lung squamous cell carcinoma (Lu Sq), pancreatic adenocarcinoma (P. Ad), prostate adenocarcinoma (Pr Ad), pheochromocytoma and paraganglioma (Ph&P), rectal adenocarcinoma (R. Ad), sarcoma (Sar.), melanoma (Mel.) thyroid carcinoma (Th C.), thymoma (Thym.), gastric adenocarcinoma (G. Ad) and endometrial carcinoma (En C.) expressed as transcripts per million (tpm) [29]. **c** SNU-1 and NUGC3 were withdrawn from growth factor stimulation by culture in growth factor-depleted calf serum as described in the Methods. Withdrawn cells were cultured in serum-free medium for two hours and then in serum-free medium in the presence of the indicated concentrations of insulin, IGF-1 or IGF-2 and lysed. Phosphorylated (Phospho.) insulin Tyr 1150/1151 and type I IGF Tyr 1135/1136 receptors (IGF receptors), Akt Ser 473, ERK1 Thr 202/204 and ERK2 Thr 185/187 were analysed by western transfer as described in the legend to Fig. 2. The relative EC_50_ values are indicated by blue lines. Asterisks indicate concentrations at which there was significantly more phosphorylated protein in the presence of ligand than in its absence (one-way ANOVA; *p* < 0.05). **d** NUGC3 cells were withdrawn from stimulatory factors in serum by culture in withdrawal medium for one day, incubated in the absence or presence of 50 ngml^−1^ insulin, IGF-1 or IGF-2 in withdrawal medium for the indicated lengths of time, lysed and DNA content measured. Asterisks indicate times at which there was significantly more DNA in the presence of ligand than in its absence (two-way ANOVA; insulin, *p* < 0.0001; IGF-1, *p* < 0.0001; IGF-2, *p* < 0.000)

Pancreatic or pharmaceutical insulins are unlikely to influence tumour progression if tumours synthesise insulin in biologically significant amounts. Relative mRNA abundance analysis demonstrated that *INS*, which encodes insulin, is not expressed in the majority, and weakly in a tiny minority, of gastric adenocarcinomas (Fig. 3a). Median relative *IGF1* mRNA abundance was around 8 (log_2_). Median relative *IGF2* mRNA abundance was higher. *INS*, *IGF1* and *IGF2* CNV and relative overexpression were detected in 13% of gastric tumours. Co-incident *INS* and *IGF2* amplification occurs because *INS* lies 10 kb upstream of *IGF2* at Chr 11p15.5. Expression of *IGF2* was, but of *INS* and *IGF1* was not, associated with CNV (Kruskal Wallis; *p* < 0.05). *INS, IGF1* and *IGF2* mRNAs were measured in a separate cohort of 1065 gastric adenocarcinomas by microarray hybridisation [28]. Low or absent expression of *INS*, higher expression of IGF-1 and even higher expression of IGF2 were confirmed.

The rarity of *INS* expression in gastric adenocarcinoma led us to investigate its expression in other adult cancers, twenty three with comparator normal tissue (Fig. 3b) and nine without (data not shown) [29]. *INS* expression was detected in pancreatic adenocarcinoma and rare neural tumours, pheochromocytoma and paraganglioma, and in their and glioblastoma and thymoma’s corresponding normal samples. Expression was highest in normal pancreas. Thus, apart from pancreatic adenocarcinoma, pheochromocytoma and paraganglioma, any direct tumour effects in other cancer types will depend upon pancreatic or pharmaceutical insulins.

### Responsiveness of gastric adenocarcinoma cells to insulin

The concentrations at which insulin activated intracellular signal transduction in serum-free medium were compared to those of IGF-1 and IGF-2. Insulin stimulated concentration-dependant autophosphorylation of the IGF receptors, phosphorylation of Akt in the PI3-kinase/Akt pathway, and increased phosphorylation of ERK1 and ERK2 in the Ras/Raf/MAPK pathway, (one-way ANOVA; *p* < 0.01) (Fig. 3c). The relative EC_50_ values for insulin-stimulated protein phosphorylation in gastric adenocarcinoma cells, 1.2–5 ngml^−1^ (0.2–2.6 nM) were similar to those of IGF-1 and IGF-2.

Proliferative effects of insulin on gastric adenocarcinoma cells were compared to those of IGF-1 and IGF-2. Over the first two days, growth rates in the absence and presence of ligand were not dissimilar due probably to the presence of residual serum-derived growth factors. Thereafter, cells grew more in the presence of insulin, IGF-1 or IGF-2 than in their absence. Growth was fivefold higher between days three and six in cells grown in the presence of insulin (two-way ANOVA; *p* < 0.0001) (Fig. 3d). Cells did not reach confluence during these experiments.

**Fig. 4:**
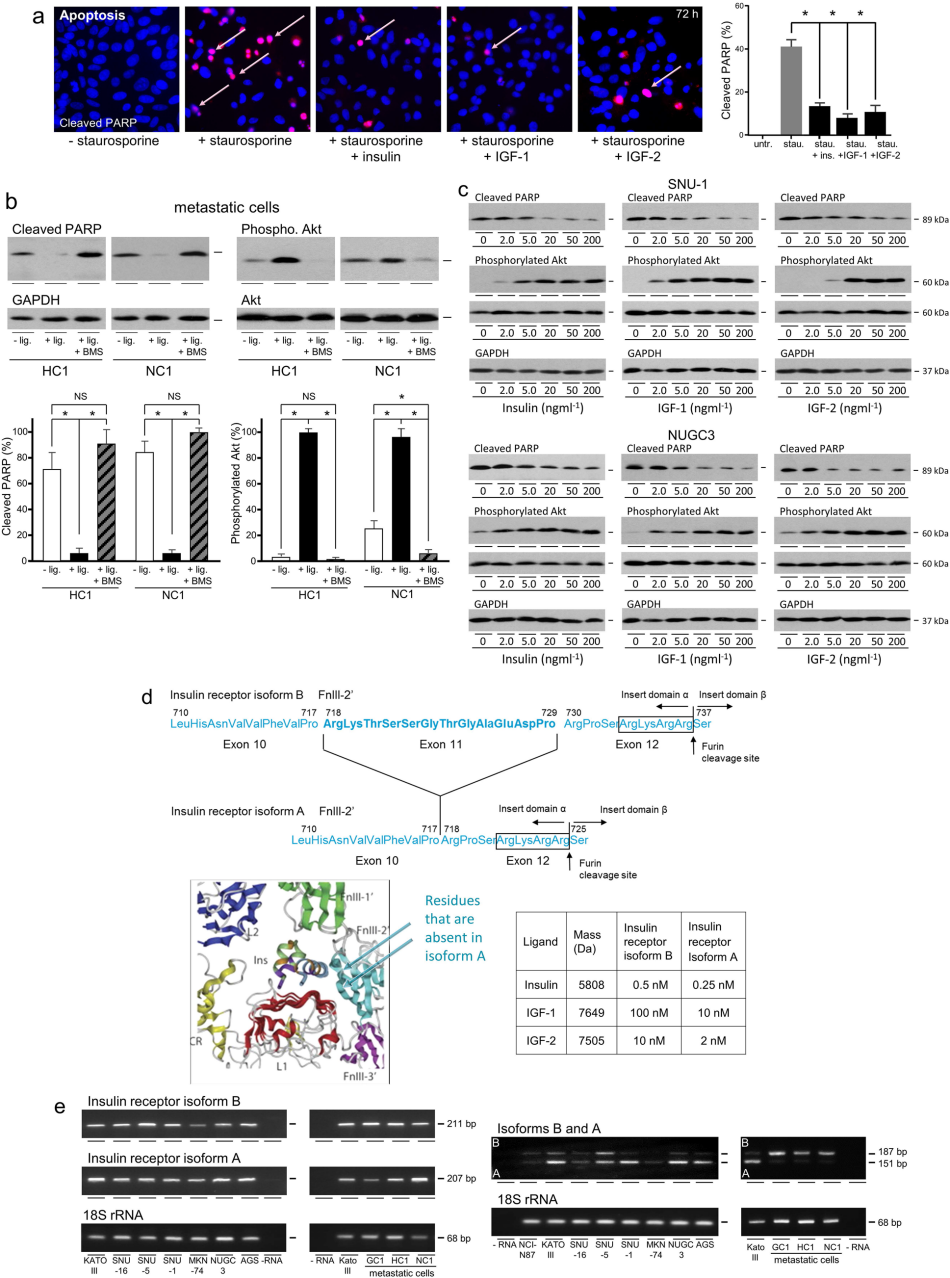
Cell survival effects of insulin, IGF-1 and IGF-2 in gastric adenocarcinoma. **a** NUGC3 were incubated in the absence or presence of 0.5 µM staurosporine without or with 50 ngml^−1^ ligand for 4 h, fixed and evidence of cell death analysed by immunofluorescent detection of cleaved Asp 214 PARP (pink arrows). The proportions of cells in which cell death had been induced are shown as means ± SEM (two-way ANOVA; *p* < 0.001). **b** Metastatic cells isolated from patients were added to poly-HEMA-coated wells in serum-free medium to prevent attachment and induce anoikis and incubated in serum-free medium in the absence or presence of ligand (lig.) minus or plus BMS-754807 for 24 h after which cleaved PARP, phosphorylated Akt Ser 473, Akt and GAPDH were measured by western transfer. Asterisks indicate if there was statistically significantly less cleaved PARP or more phosphorylated Ser 473 Akt after incubation in the presence of ligand, or more cleaved PARP or less phosphorylated Ser 473 Akt after incubation in the presence of ligand and BMS-754807, than in the presence of ligand alone (two-way ANOVA; *p* < 0.01). NS indicates data are not significantly different. **c** SNU-1 and NUGC3 were incubated with staurosporine and different concentrations of ligand prior to analysis of cleaved PARP, phosphorylated Akt Ser 473, Akt and GAPDH by western transfer. **d** The primary sequences of the insulin receptor in the second fibronectin type III domain (FnIII-2’) that differentiate isoform B from isoform A are shown (adapted from [10]). Insulin is shown positioned in the binding pocket of isoform B with the amino acid residues in FnIII-2’encoded by exon 11 that are absent in isoform A arrowed (adapted from [19]). The molecular masses of the IGF ligands and their affinities for insulin receptor isoforms B and A are listed [10]. Please see electronic supplementary material for further details (online resource 1). **e** RNA extracted from gastric cancer cells and metastatic patient samples incubated in untreated FCS-containing medium was reverse transcribed and cDNA amplified with primers designed to detect insulin receptor isoform B (211 bp), insulin receptor isoform A (207 bp), isoform B and isoform A simultaneously (187 bp and 151 bp, respectively) or 18S rRNA (68 bp)

To investigate effects of insulin on cell survival, caspase-dependent apoptosis was induced with the kinase inhibitor staurosporine and cleaved PARP visualised in apoptotic nuclei by immunofluorescence (Fig. 4a). Cleaved PARP was detected in a smaller proportion of nuclei after incubation in the presence of insulin, indicative of protection against cell death. Similar levels of protection were effected by IGF-1 and IGF-2.

Metastatic gastric adenocarcinoma cells were protected by ligand against anoikis, which is programmed cell death induced by loss of cell attachment, in serum-free medium (Fig. 4b). This ligand-dependent protection was prevented by BMS-754807 in serum-free medium (two-way ANOVA; *p* < 0.001). Inhibition and activation of the PI3-kinase/Akt pathway, which is required for protection of gastric cancer cells from cell death [7], accompanied ligand-dependent protection against cell death and its prevention. Ligand-dependent protection of SNU-1 and NUGC3 from anoikis in serum-free medium, and from  apoptosis, was inhibited also by BMS-754807 (data not shown).

Incubation of gastric adenocarcinoma cells with different insulin, IGF-1 or IGF-2 concentrations prior to induction of apoptosis demonstrated concentration-dependent protection (Fig. 4c). Less cleaved PARP was detected at 5 ngml^−1^ insulin, IGF-1 and IGF-2 and above. Activation of the PI3-kinase/Akt pathway was detectable at 2 ngml^−1^ ligand and above.

The concentrations at which insulin, IGF-1 and IGF-2 activated signal transduction and stimulated cell proliferation and survival indicated that activity might be via their cognate receptors. However, insulin receptor isoform A lacks twelve amino acids encoded by exon 11 [20] that occupy space in the insulin receptor ligand binding pocket of isoform B (Fig. 4d) [10, 30]. More capacious in their absence, isoform A’s ligand binding pocket accommodates better, and has a higher affinity for, the larger IGF-1 and IGF-2 molecules [10, 19]. Please see online resource 1 for details of affinities for hybrid receptors.

Amplification of insulin receptor transcripts by RT-PCR detected both isoform B and isoform A mRNAs in all gastric adenocarcinoma cell lines and metastatic cells (Fig. 4e). Expression of isoform A means that IGF-1 and IGF-2 will activate the insulin receptor at lower concentrations than if only isoform B was expressed. Simultaneous amplification of both isoform mRNAs indicated that isoform A was relatively more abundant in some gastric adenocarcinoma cells, whereas isoform B was relatively more abundant in metastatic cells isolated from patients (Fig. 4e).

### Insulin receptor knockdown reduces growth and induces cell death

**Fig. 5:**
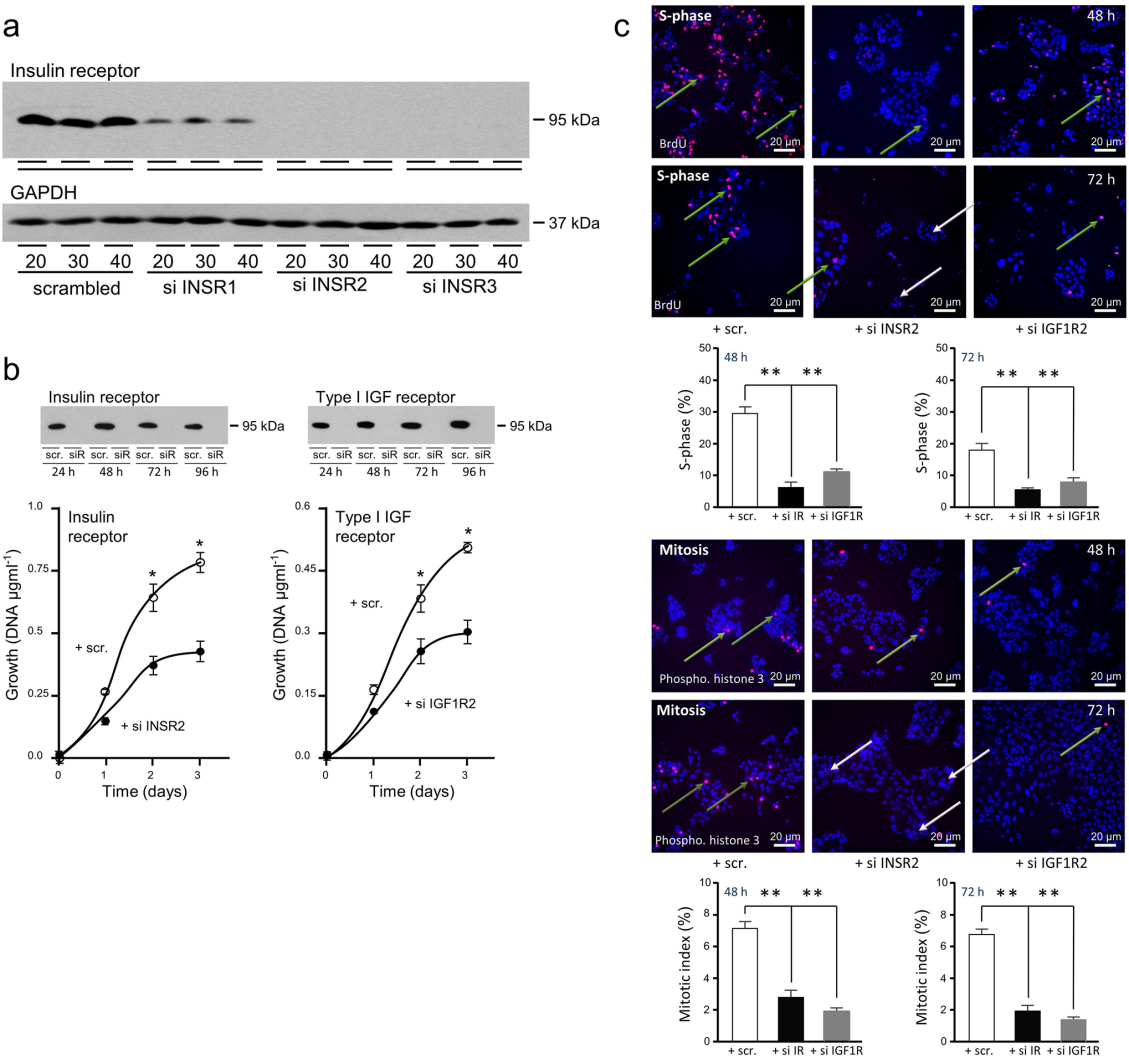
Knockdown of the insulin receptor reduces gastric cancer cell growth and division. **a** NUGC3 cells were transfected with scrambled oligonucleotide, siINSR1, siINSR2 or siINSR3, cultured for three days in full, untreated FCS-containing medium after which insulin receptor levels were analysed by western transfer. **b** NUGC3 cells were transfected with scrambled oligonucleotide (scr.), siINSR2 (siR) or siIGF1R2 (siR) and cultured in full, untreated FCS-containing medium for the indicated lengths of time prior to analysis of receptor expression or DNA content. Asterisks indicate times at which there was significantly less DNA after transfection with siINSR2 or siIGF1R2 than with scrambled oligonucleotide (two-way ANOVA; *p* < 0.0001). **c** NUGC3 cells were transfected with scrambled oligonucleotide (scr.), siINSR2 (si IR) or siIGF1R2 (si IGF1R), plated onto coverslips, cultured in full, untreated FCS-containing medium for three days and assayed for BrdU incorporation (green arrows) or histone H3 Ser 10 phosphorylation (khaki arrows). Asterisks indicate if the proportion of cells in either phase of the cell cycle is significantly lower after transfection with siINSR2 or siIGF1R2 than with the scrambled oligonucleotide (one-way ANOVA; *p* < 0.0001). Pale pink arrows indicate apoptotic cells

The individual activities of the insulin and type I IGF receptors were tested by genetic knockdown. First, effective knockdown of insulin receptor was demonstrated (Fig. 5a). Insulin receptor knockdown reduced untreated FCS-stimulated growth of gastric adenocarcinoma cells (Fig. 5b). Similar effects of type I IGF receptor knockdown replicated previous results [7]. Images above the growth curves illustrate that receptor knockdown was maintained throughout the experiments.

The effects of receptor knockdown on cell cycle progression were tested. Analysis of BrdU incorporation into newly synthesised DNA demonstrated that 30% of cells cultured in untreated FCS-containing medium were in S-phase (Fig. 5c). Knockdown of the insulin receptor and type I IGF receptor [7] reduced the proportion of cells in S-phase two- to fourfold after two and three days, respectively. Similarly, the proportion of mitotic cells was reduced after knockdown of either receptor (One-way ANOVA; *p* < 0.0001).

**Fig. 6:**
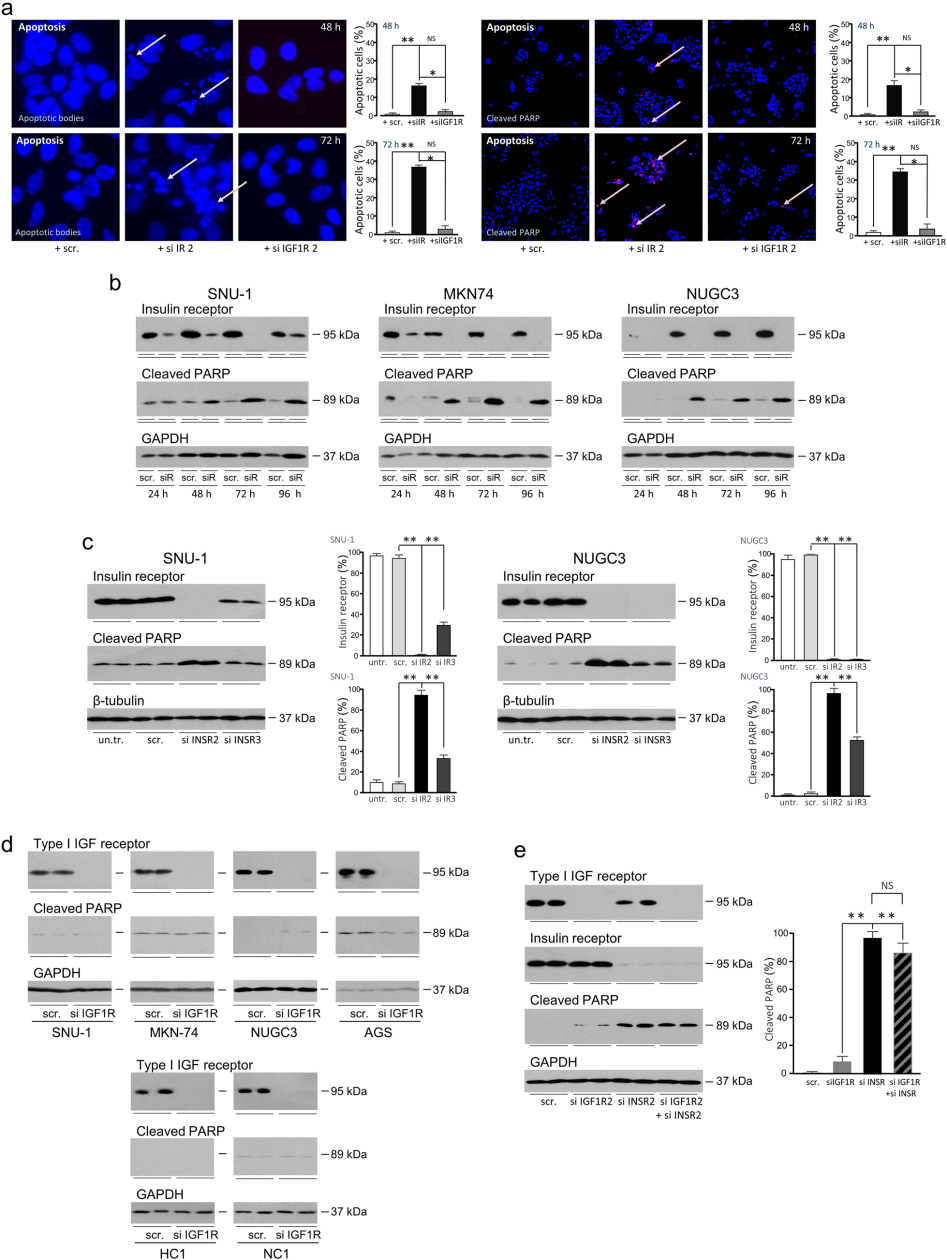
Knockdown of the insulin receptor induces cell death in gastric cancer cells. **a** NUGC3 were transfected with scrambled oligonucleotide (scr.), siINSR2 or siIGF1R2 and analysed as described in the legend to Fig. 5. High magnification images facilitate visualisation of apoptotic nuclei (pale pink arrows). Cells were analysed next for the presence of cleaved PARP (pink arrows). Asterisks indicate that the proportion of apoptotic cells, or cells with cleaved PARP, is significantly higher after transfection with siINSR2 than with the scrambled oligonucleotide (**) or siIGF1R2 (*) (one-way ANOVA; *p* < 0.0001). NS indicates data are not significantly different. **b** SNU-1, MKN-74 and NUGC3 cells were transfected with scrambled oligonucleotide (scr.) or siINSR2 (siR) and cultured as indicated in untreated-FCS-containing medium prior to analysis of insulin receptor, cleaved PARP or GAPDH by western transfer. **c** SNU-1 and NUGC3 were transfected with scrambled oligonucleotide, siINSR2 or siINSR3 and cultured for three days in untreated-FCS-containing medium prior to measurement of insulin receptor, cleaved PARP or β-tubulin by western transfer. Asterisks (**) indicate that there was significantly less insulin receptor or more cleaved PARP after transfection with siINSR2 or siINSR3 than with scrambled oligonucleotide (one-way ANOVA; *p* < 0.001). **d** SNU-1, MKN-74, NUGC3 and AGS, and patient samples, HC1 and NC1, were transfected with scrambled oligonucleotide (scr.) or siIGF1R2, cultured for three days in untreated-FCS-containing medium and type I IGF receptor, cleaved PARP and GAPDH analysed. **e** NUGC3 were transfected with scrambled oligonucleotide (scr.), siINSR2, siIGF1R2 or both siINSR2 and siIGF1R2 and cultured in untreated-FCS-containing medium for three days prior to measurement of type I IGF receptor, insulin receptor, cleaved PARP or GAPDH by western transfer. There was significantly more cleaved PARP after transfection with siINSR2, or siINSR2 and siIGF1R2 (one-way ANOVA; *p* < 0.001)

Fragmented nuclei of apoptotic cells discernible after transfection with siINSR2 (Fig. 5c) were clearly visible in higher magnification images (Fig. 6a). Thirty per cent of cells appeared apoptotic after 72 h. Apoptotic nuclei were absent in cells transfected with scrambled siRNA and rare after transfection with siIGF1R2. That apoptosis was initiated by insulin receptor but not by type I IGF receptor knockdown was confirmed by detection of cleaved PARP specifically in nuclei of cells transfected with siINSR2 (One-way ANOVA; *p* < 0.0001).

The cell survival activity of the insulin receptor was corroborated by transfection of SNU-1, MKN-74 and NUGC3 with siINSR2. PARP cleavage was induced after 48 h and persisted up to 96 h after transfection (Fig. 6b). Similar results were obtained with siINSR3 (Fig. 6c). In contrast, knockdown of the type I IGF receptor by transfection of SNU-1, MKN-74, NUGC3 or AGS with siIGF1R2 did not induce PARP cleavage (Fig. 6d). Equally, apoptosis was not induced by type I IGF receptor abrogation in metastatic cells isolated from patients.

The effects of simultaneous knockdown of the receptors was investigated. Transfection with siIGF1R2 reduced type I IGF receptor but not insulin receptor levels (Fig. 6e). Transfection with siINSR2 reduced specifically insulin receptor levels. More programmed cell death was induced by insulin receptor knockdown than by type I IGF receptor knockdown. Combined knockdown was no more effective than knockdown of the insulin receptor alone.

## Discussion

Our finding that higher tumour *INSR* expression is associated with shorter overall patient survival concurs with a report that membranous insulin receptor is associated with shorter tumour-specific survival [31]. A trend towards an association between endothelial cell insulin receptor and survival was noted; single-cell transcriptome analysis could elucidate this observation. That patients whose tumour cells have higher *INSR* expression, and a priori higher insulin receptor levels, have worse survival implies that insulin promotes gastric cancer. Higher tumour cell insulin receptor concentration and availability should allow cells to achieve higher receptor occupancy and hence increased responsivity to insulin.

The association between higher *IGF1R* expression and shorter overall patient survival agrees with a previous study [32]. Higher *IGF1R* expression is associated also with shorter overall survival in paediatric but not adult glioma [33].

Pharmacological inhibition was effective in gastric adenocarcinoma cells without amplification of *ERBB2*, *FGFR2* or *MET*. Cell growth and mitosis were inhibited, and programmed cell death was initiated in the presence of the plethora of growth and survival factors that are present in untreated FCS. These findings are consistent with the insulin and IGF pathway having a significant role in triple-negative gastric adenocarcinoma cell proliferation and survival.

The present study focused on the 70% of gastric adenocarcinomas without amplification of *ERBB2*, *FGFR2* or *MET*. Those with amplification and overexpression are predicted to be driven predominantly by signal via the tyrosine kinase receptor encoded by the amplified oncogene. Nevertheless, NCI-N87, Kato III, SNU-16 and SNU-5 respond to IGF stimulation [7] and BMS-754807 reduced NCI-N87 growth (Fig. 1g). Insulin and IGF signal transduction in these cells, which have high insulin receptor levels, may be able to sustain growth and survival if the dominant receptor tyrosine kinase is targeted. Sensitivity to inhibition, and activity, of the insulin and IGF signal transduction pathway has been demonstrated in lapatinib- and trastuzumab-resistant NCI-N87 [34, 35]. Further, insulin and type I IGF receptors mediated lapatinib resistance of *HER2*-amplified SNU-216 gastric cancer cells [34].

Calle et al. [9] concluded that increased levels of endogenous hormones associated with obesity, such as insulin, have a more profound effect on cancer death rates than they do on incidence. This is the first report of direct effects of insulin on gastric adenocarcinoma cells. Insulin activated signal transduction, stimulated cell growth and increased cell survival. *INS* was not expressed in most gastric adenocarcinomas, or in the majority of other adult cancers types. It can be inferred that direct cancer-promoting effects of insulin on tumour cells depend upon endocrine or therapeutic sources.

The relative EC_50_ insulin concentrations in gastric adenocarcinoma cells were 1.2–15 ng/ml (0.2–2.6 nM). These concentrations are above the relatively low fasted plasma insulin concentrations of leaner individuals (0.1–0.52 ng/ml (18–90 pM) [36]. But they overlap both the slightly higher fasted plasma insulin concentrations, and non-fasted concentrations, in the general population, 0.01–4.5 ng/ml (1.8–767.4 pM) and 0.09–8.7 ng/ml (15.9–1365.9 pM), respectively [13].

Insulin-resistant individuals with compensatory hyperinsulinemia have high plasma insulin concentrations, 0.6–25 ng/ml (0.1–4.3 nM) [36, 37]. In patients with type II diabetes, therapeutic insulin concentrations reach 30 ng/ml (5.2 nM) [38]. Thus some individuals in the general population, those with weight gain or hyperinsulinemia, and those who inject insulin, have plasma insulin concentrations at or above the EC_50_ insulin concentrations effective in gastric adenocarcinoma cells. Our findings could explain why overweight and obese individuals, and type I and type II diabetics, are more likely to present with symptomatic gastric cancer [12, 17, 39], and why obese men have twice the death rate from gastric cancer as lean individuals [9].

High tumour *IGF1* and *IGF2* expression, *IGF2* is the 25th most over-expressed gene in gastric adenocarcinoma [29], implies local IGF-1 and IGF-2 synthesis. Tumour synthesis coupled with demonstration that IGF-1 and IGF-2 stimulate gastric adenocarcinoma cell growth and survival [7, 40], suggests potential autocrine activity. Autocrine activities have been reported for IGF-1 [41, 42] and IGF-2 [40, 41, 43]. Twenty-five gastric adenocarcinomas had relative IGF-2 mRNA abundances ≥ 20,000 (14.29 log_2_) of which only three had concurrent gene amplification. In the other cases, another mechanism such as loss of *IGF2* imprinting, [44] or enhancer hijacking by tandem duplication [45] must account for the overexpression.

Genetic knockdown demonstrated that both receptors transmit mitogenic signals in gastric adenocarcinoma. This is the first report of a cytostatic effect of *INSR* knockdown. The cytostatic effect of *IGF1R* knockdown agrees with previous findings in gastric, breast and pancreatic cancer [7, 23, 46].

Demonstration that the insulin receptor transmits a strong cell survival stimulus in gastric adenocarcinoma was unexpected. In a transgenic model of pancreatic neuroendocrine carcinogenesis and an oestrogen-unresponsive breast cancer cell line, insulin receptor knockdown had no effect alone but sensitised cells to pharmacological inhibition of the type I IGF receptor [47].

Dependence of gastric adenocarcinoma cell survival upon insulin receptor but not type I IGF receptor indicates that IGF-1 and IGF-2 promote cell survival through the insulin receptor. Expression of insulin receptor isoform A, for which IGF-1 and IGF-2 have higher affinities than they have for isoform B, [10, 30] by gastric adenocarcinoma cells makes this supposition plausible. Relatively higher expression of insulin receptor isoform B, for which insulin has considerably higher affinity than IGF-1 or IGF-2 has, was apparent in metastatic gastric adenocarcinoma cells. A corollary is that preferential responsiveness to insulin is predicted in these metastatic cells.

Our findings support the importance of interventions to lower plasma insulin levels in diabetics with cancer, and to reduce weight and consequent hyperinsulinemia, in overweight and obese cancer patients. Future work should investigate the dual roles of insulin and its receptor in other cancer types associated with obesity, diabetes mellitus or high insulin states [9, 10, 48].

## Supplementary Information

Below is the link to the electronic supplementary material.

Supplementary file1 (PDF 146 KB)
