# Supplementary material for: Insulin and the insulin receptor collaborate to promote human gastric cancer
Source: Gastric Cancer. 2021 Sep 23;25(1):107–23. doi: 10.1007/s10120-021-01236-y (PMC8732810; doi:10.1007/s10120-021-01236-y)
Supplement: Supplementary file 1 — Supplementary file1 (PDF 146 KB) [file 10120_2021_1236_MOESM1_ESM.pdf]

Supplementary Table. Affinities of insulin, IGF-1 and IGF-2 for all possible homodimers and heterodimers between isoform A (IR-A) and isoform B (IR-B) of the insulin receptor and the type I IGF receptor (IGF-IR).

Ligand	IR-B:IR-B	IR-A:IR-A	IR-B:IR-A	IGF-1R:IGF-1R	IR-B:IGF-1R	IR-A:IGF-1R
Insulin	0.5 nM$	0.25 nM	0.5 nM	100 nM	80 nM	70 nM*
IGF-1	100 nM	10 nM	40 nM	0.2 nM	0.3 nM	0.4 nM
IGF-2	10 nM	2 nM	10 nM	0.5 nM	0.5 nM	0.7 nM

$ Affinities [1] were taken from or estimated from original sources. [2-7] *The affinities measured by different methods for the heterodimer between isoform A of the insulin receptor and the type 1 IGF receptor are somewhat discrepant.

Supplementary References

1. Westley RL, May FEB. A Twenty-First Century Cancer Epidemic Caused by Obesity: The Involvement of Insulin, Diabetes, and Insulin-Like Growth Factors. International Journal of Endocrinology. 2013.

2. Frasca F, Pandini C, Scalia P, Sciacca L, Mineo R, Costantino A, et al. Insulin receptor isoform A, a newly recognized, high-affinity insulin-like growth factor II receptor in fetal and cancer cells. Molecular and Cellular Biology. 1999;19(5):3278-88.

3. Belfiore A, Malaguarnera R. Insulin receptor and cancer. Endocr Relat Cancer. 2011;18(4):R125-47.

4. Benyoucef S, Surinya KH, Hadaschik D, Siddle K. Characterization of insulin/IGF hybrid receptors: contributions of the insulin receptor L2 and Fn1 domains and the alternatively spliced exon 11 sequence to ligand binding and receptor activation. Biochem J. 2007;403(3):603-13.

5. Yamaguchi Y, Flier JS, Benecke H, Ransil BJ, Moller DE. Ligand-binding properties of the two isoforms of the human insulin receptor. Endocrinology. 1993;132(3):1132-8.

6. Pandini G, Vigneri R, Costantino A, Frasca F, Ippolito A, Fujita-Yamaguchi Y, et al. Insulin and insulin-like growth factor-I (IGF-I) receptor overexpression in breast cancers leads to insulin/IGF-I hybrid receptor overexpression: evidence for a second mechanism of IGF-I signaling. Clin Cancer Res. 1999;5(7):1935-44.

7. Pandini G, Frasca F, Mineo R, Sciacca L, Vigneri R, Belfiore A. Insulin/insulin-like growth factor I hybrid receptors have different biological characteristics depending on the insulin receptor isoform involved. J Biol Chem. 2002;277(42):39684-95.
